# Case report: Parvovirus B19 encephalitis following rituximab therapy in a patient with immune-mediated thrombocytopenia

**DOI:** 10.3389/fimmu.2026.1698621

**Published:** 2026-04-17

**Authors:** Valerio Campolo, Martin Kitzrow, Guglielmo Lucchese, Dietrich Sturm

**Affiliations:** 1 Department of Neurology, Agaplesion Bethesda Hospital, Wuppertal, Germany; 2 Department of Psychiatry, Psychotherapy and Psychosomatics, Psychiatry University Hospital Zurich, Zurich, Switzerland; 3 Department of Experimental Medicine, University of Salento, Lecce, Italy; 4 Department of Neurology, “Vito Fazzi” University Hospital, Lecce, Italy; 5 Department of Neurology, University Medicine Greifswald, Greifswald, Germany

**Keywords:** IMT, ITP (idiopathic thrombocytopenic purpura), PVB19, RTX (rituximab), seizure

## Abstract

Parvovirus B19 infection is mostly asymptomatic in immunocompetent individuals but may cause severe manifestations when humoral immunity is impaired. Anti-CD20 therapies such as rituximab induce prolonged B-cell depletion and hypogammaglobulinemia, affecting the immune coordination and lowering the threshold for viral neuroinvasion and persistence within the central nervous system (CNS). We present a case of a 61-year-old patient who developed new-onset seizures 9 months after completing rituximab as fourth-line therapy for immune-mediated thrombocytopenia. Brain MRI showed a focal hyperintense lesion in the left peri-insular region with edema. Parvovirus B19 DNA was detected in the cerebrospinal fluid via polymerase chain reaction, while extensive testing for other infectious and autoimmune causes of encephalitis was negative. Immunological work-up demonstrated persistent depletion of CD19^+^ B lymphocytes and severe hypogammaglobulinemia. This case highlights the role of impaired humoral immunity in facilitating viral persistence within the CNS and emphasizes the need to consider parvovirus B19 and other uncommon pathogens as a potential cause of encephalitis in patients with prior anti-CD20 therapy, even months after treatment completion.

## Background

Primate erythroparvovirus 1, also referred to as parvovirus B19, belongs to the family *Parvoviridae*, genus *Erythroparvovirus*, and is one of the smallest non-enveloped DNA viruses (1). It is mostly known to cause infections in the pediatric population; however, it can also affect adults. In immunocompromised hosts, the most common consequences are pure red-cell aplasia and arthritis (1). Immune thrombocytopenia (ITP) is an acquired disease caused by an autoimmune reaction against platelets and megakaryocytes. Steroids are the first-line treatment, if necessary, in combination with intravenous immunoglobulins (IVIGs). Second-line therapies include rituximab, thrombopoietin receptor agonists (TRAs), or splenectomy, depending on patient age, preferences, and clinical scenario (2). Rituximab, a chimeric monoclonal antibody targeting CD20-expressing B lymphocytes, is widely used in autoimmune diseases as well as hematological disorders. While generally well-tolerated, rituximab induces prolonged depletion of CD20-expressing B lymphocytes, which may persist for months after treatment completion (3, 4). This results in impaired humoral immune responses, reduced generation of neutralizing antibodies, and, in a subset of patients, secondary hypogammaglobulinemia. As a consequence, viral clearance may be compromised, favoring viral persistence or reactivation. In the context of anti-CD20 therapy, reactivation or chronic infection has been well documented for hepatitis B and herpes viruses, highlighting the critical role of B cell-mediated immunity in long-term viral control (7). In contrast, data on parvovirus B19-associated central nervous system (CNS) disease remain scarce (3, 4).

## Case history

**Figure 1:**
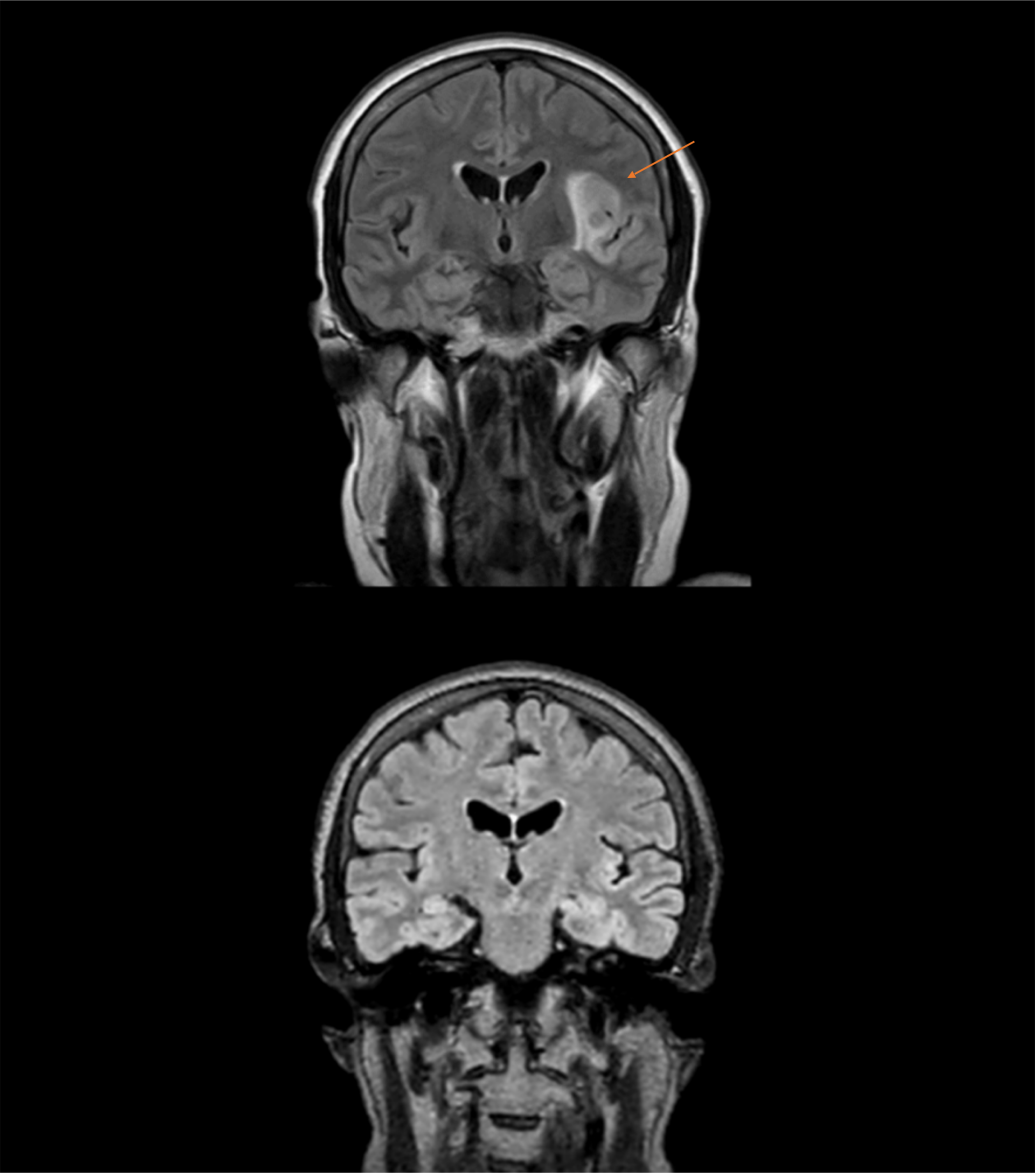
Brain MRI (1.5 Tesla, slice thickness 5 mm) with fluid-attenuated inversion recovery (FLAIR) sequence demonstrating a left-hemispheric peri-insular lesion (red arrow) and complete regression of the lesion at 3-month follow-up.

A 61-year-old female patient with no history of epilepsy was admitted due to a focal epileptic seizure with self-limiting secondary generalization. The patient had a long-standing history of chronic immune-mediated thrombocytopenia, diagnosed 16 years ago and initially refractory to corticosteroid therapy, but spontaneously converted to a chronic form. After relapse with severe thrombocytopenia 8 years later, repeated high-dose dexamethasone pulse therapy (40 mg/day for 4 days) failed to induce a sustained response, and second-line treatment with the TRA eltrombopag was initiated and continued for 4 years. A further relapse led to third-line therapy with romiplostim, without durable platelet recovery. Consequently, fourth-line therapy with rituximab was initiated at standard dosing (375 mg/m^2^ body surface area), leading to a cumulative dose of 2.4 g. At admission, our patient showed no significant focal neurological deficit. The initial brain CT and laboratory tests did not indicate any acute pathology. The patient was immediately treated with levetiracetam (1 g/day). Nevertheless, additional seizures occurred, mostly focal, with prolonged speech impairment. However, MRI showed circumscribed areas of increased white matter intensity in the left peri-insular region on T2 and fluid-attenuated inversion recovery (FLAIR) images, without gadolinium enhancement (**Figure 1**). EEG showed left-sided centroparietal region dysfunction. Finally, based on cerebrospinal fluid (CSF) analysis, we diagnosed a focal encephalitis of the left insular region. CSF analysis showed lymphocytic pleocytosis (50 cells/µL) and elevated protein concentration (0.72 g/L). Intrathecal IgM synthesis was detectable (5.38 mg/dL, reference < 0.1), while oligoclonal bands were absent. Parvovirus B19 IgG antibodies in the CSF were strongly positive (index 20.0; reference < 0.9), with negative IgM, consistent with prior exposure rather than acute primary infection. Detection of parvovirus B19 DNA in the CSF via polymerase chain reaction (PCR) proved an active CNS infection. Furthermore, extended serological and CSF analyses were performed. Serum immunoglobulin testing, at the time of diagnosis, confirmed severe hypogammaglobulinemia, with markedly reduced IgG (279 mg/dL; reference 700–1,600 mg/dL) and low IgA levels (11 mg/dL; reference 70–400), while IgM was markedly reduced (18 mg/dL; reference 40–230 mg/dL) (**Table 1**). Microbiological testing excluded alternative infectious etiologies (negative PCR testing of CSF for herpes simplex virus types 1 and 2, varicella zoster virus, cytomegalovirus, Epstein–Barr virus, enteroviruses, JC virus, *Mycoplasma pneumoniae*, and *Toxoplasma gondii*). Furthermore, there were no findings for the presence of autoimmune encephalitis. Following initial empirical anti-infective treatment with aciclovir, the patient received anti-edematous therapy with dexamethasone. Because of additional focal seizures with speech arrest, a higher dose of levetiracetam (3 g/day) was administered, and add-on treatment with lacosamide (100 mg/day) was initiated. As the patient did not develop any further seizures during the course of treatment and the known IgA deficiency persisted, additional treatment with IVIGs was not administered, as IgA deficiency is associated with more frequent adverse drug reactions. In a follow-up examination 3 months later, neuropsychological tests revealed mild impairments in attention and concentration, but an MRI scan showed full recovery (**Figure 1**). A chronological sequence of the entire case is shown in (**Table 2**).

**Table 1:** Relevant results of serum and flow cytometry analysis.

CD19^+^ B lymphocytes	13/µL (132–422)
Immunoglobulin G	279 mg/dL (700–1,400)
Immunoglobulin M	18 mg/dL (40–230)
Immunoglobulin A	11 mg/dL (70–400)

Normative values in parentheses.

**Table 2:** Time course of the individual medical history presented, including rituximab exposure, neurological presentation, diagnostic work-up, immunological findings, treatment, and follow-up.

Time point	Phase	Key findings
T0–T1	Rituximab exposure and immunosuppression	Rituximab for ITP (4th line), completed 9–10 months earlier; probable persistent CD19^+^ B-cell depletion and hypogammaglobulinemia.
T2 (after 10 months)	Neurological onset and diagnostics	Focal epileptic seizures with secondary generalization and speech impairment; MRI peri-insular FLAIR lesion without enhancement; EEG region dysfunction; CSF pleocytosis and parvovirus B19 PCR positive.
T3	Immunological work-up	Depletion of CD19^+^ B lymphocytes (13/µL) and serum IgG (279 mg/dL), IgM (18 mg/dL), and IgA (11 mg/dL).
T4 (>1 year)	Therapy and follow-up	Levetiracetam (up to 3 g/day), lacosamide add-on, dexamethasone; 3 months later: improvement in MRI findings, mild neuropsychological attention deficit, no epileptic seizures.

ITP, immune thrombocytopenia; FLAIR, fluid-attenuated inversion recovery; CSF, cerebrospinal fluid.

## Discussion

The case we present vividly describes acute infectious focal encephalitis due to parvovirus B19 as a complication of rituximab therapy. Parvovirus B19 exhibits a pronounced tropism for erythroid progenitor cells through interaction with the P antigen (globoside Gb4Cer), which mediates viral entry (5). Nevertheless, during acute infection, the virus reaches high circulating titers, allowing dissemination to multiple organs, and the expression of globoside and accessory entry factors on endothelial, synovial, myocardial, and selected neural cells provides a plausible route for infection of non-erythroid tissues (6). In these compartments, cellular permissiveness for complete viral replication is generally limited, resulting in abortive or low-level infection rather than productive virion assembly. Despite this restriction, the expression of the non-structural protein NS1 has been shown to induce cellular DNA damage, apoptosis, and pro-inflammatory signaling, including the activation of NF-κB-dependent pathways, thereby establishing a mechanistic link between viral persistence and tissue inflammation even in the absence of robust viral replication (6, 7). Consistent with this model, parvovirus B19 DNA has been detected in the myocardium and central nervous system tissue long after primary infection, often without concomitant viremia or serological evidence of ongoing infection (8, 9). In immunocompetent hosts, such persistence is typically clinically silent and controlled by coordinated humoral and cellular immune responses. In contrast, the depletion of CD20-positive B cells profoundly impairs neutralizing antibody production, a key mechanism for viral clearance from the circulation, thereby facilitating prolonged or recurrent viremia and repeated seeding of peripheral tissues (10). In this setting, viral persistence may be sustained not only by continued replication in erythroid progenitors but also by the inability to eliminate low-level infection within non-erythroid cells, where antibody-mediated neutralization is intrinsically less effective. Importantly, this state does not necessarily result in uncontrolled viral replication but may instead promote a smoldering infection characterized by intermittent antigen expression and chronic immune activation (10). Superimposed immune reconstitution phenomena, such as partial recovery of cellular immune responses after prolonged B-cell depletion or immune modulation by corticosteroids, may unmask previously tolerated viral antigens and amplify local inflammatory responses, particularly within immune-privileged compartments such as the CNS. This integrated mechanism provides a plausible pathophysiological framework for parvovirus B19 encephalitis occurring in patients with sustained hypogammaglobulinemia following anti-CD20 therapy (11). The present case illustrates these mechanisms in a clinically relevant manner. Despite strongly positive parvovirus B19 IgG antibodies, serological findings were considered of limited diagnostic value due to profound hypogammaglobulinemia and persistent B-cell depletion. In contrast, detection of parvovirus B19 DNA in the CSF via PCR provided decisive evidence of active CNS infection, underscoring the importance of molecular diagnostics in immunocompromised patients. Additional CSF findings, including intrathecal IgM synthesis, supported active intrathecal immune activation and inflammatory encephalitis, while extensive testing excluded autoimmune encephalitis, progressive multifocal leukoencephalopathy, and alternative infectious etiologies. Only a few cases of parvovirus B19-associated neurological disease have been reported in patients treated with anti-CD20 monoclonal antibodies, including rituximab and ocrelizumab (12). Compared with previously published cases, the present report is notable for the long latency between rituximab exposure and the onset of encephalitis, as well as for the focal and self-limited clinical course. These observations suggest that parvovirus B19-associated CNS disease may occur even months after completion of B cell-depleting therapy and may remain clinically subtle in the absence of severe systemic manifestations. Currently, no causal antiviral therapy for parvovirus B19 infection has been established. IVIGs are considered the main therapeutic option in severe or progressive disease, particularly in patients with chronic viremia or hematological complications. In the present case, the decision not to administer intravenous immunoglobulin therapy was primarily based on the mild and non-progressive clinical course, early radiological improvement, and stable neurological status. The presence of severe IgA deficiency was considered an additional factor supporting a cautious therapeutic strategy because of the increased risk of adverse reactions to immunoglobulin preparations. Nevertheless, the therapeutic decision was mainly guided by the favorable clinical evolution rather than by IgA deficiency alone.

Our report has several limitations inherent to single case descriptions. First, quantitative viral load measurements for parvovirus B19 in cerebrospinal fluid were not available. Consequently, viral activity could only be inferred from qualitative PCR positivity, and it was not possible to distinguish between active viral replication and detection of residual viral DNA. Second, longitudinal data on B-cell reconstitution following rituximab therapy were not present, limiting correlation between immune recovery and symptom onset. Third, although CSF-PCR positivity strongly supports a CNS infection, histopathological confirmation was not obtained.

Nevertheless, from a clinical perspective, this case highlights the importance of long-term immunological monitoring in patients receiving rituximab and other B cell-depleting agents. In patients presenting with encephalitis of unknown origin after anti-CD20 therapy, parvovirus B19 should be considered in the diagnostic work-up, and PCR-based testing of CSF should be prioritized.

## Data Availability

The original contributions presented in the study are included in the article/supplementary material. Further inquiries can be directed to the corresponding author.
